# Sexual dimorphism in the relationship between *Forkhead-Box P2* and BMI with cognitive deficits in schizophrenia

**DOI:** 10.3389/fnagi.2022.920352

**Published:** 2022-08-03

**Authors:** Mi Yang, Ying Cui, Mei Xue, Mattew T. Forster, Xiaoe Lang, Meihong Xiu, Zezhi Li, Xiangyang Zhang

**Affiliations:** ^1^The Fourth Hospital of Chengdu, Chengdu, China; ^2^Qingdao Mental Health Center, Qingdao, China; ^3^Department of Psychiatry and Behavioral Sciences, University of Texas Health Science Center at Houston, Houston, TX, United States; ^4^Department of Psychiatry, First Hospital/First Clinical Medical College of Shanxi Medical University, Taiyuan, China; ^5^Beijing HuiLongGuan Hospital, Peking University, Beijing, China; ^6^Department of Psychiatry, The Affiliated Brain Hospital of Guangzhou Medical University, Guangzhou, China; ^7^Guangdong Engineering Technology Research Center for Translational Medicine of Mental Disorders, Guangzhou, China; ^8^Department of Psychology, University of Chinese Academy of Sciences, Beijing, China

**Keywords:** sexual dimorphism, *FOXP2*, BMI, cognitive deficit, schizophrenia

## Abstract

*FOXP2*, cognitive deficits, and schizophrenia are associated with neurodegenerative pathophyisiology. Mounting evidence suggests that body mass index (BMI) and *FOXP2* may contribute to cognitive deficits in schizophrenia. However, the sex difference in the contribution of *FOXP2* and BMI, as well as their potential interaction with cognitive deficits in schizophrenia, have not been investigated. A total of 867 schizophrenia patients and 402 controls were recruited. Cognitive function was assessed using the Repeatable Battery for the Assessment of Neuropsychological Status (RBANS). The polymorphism rs10447760 of the *FOXP2* gene was genotyped. Male schizophrenia patients had superior language performance compared to female patients (*F* = 17.83; *p*_Bonferroni_ < 0.0001). BMI was positively associated with language scores in male patients with schizophrenia (*ß* = 0.60, *t* = 3.30, *p* = 0.001), as well as in patients with schizophrenia who carried the *FOXP2* rs10447760 CC genotype (*ß* = 0.53, *t* = 3.16, *p* = 0.002). Interestingly, this association was only found in male patients with schizophrenia who also carried the *FOXP2* rs10447760 CC genotype (*ß* = 0.63, *t* = 3.44, *p* = 0.001). Our study reveals a sex difference in the language deficits of schizophrenia patients and shows sexual dimorphism in the contribution of *FOXP2*, BMI, and their interaction to cognitive deficits in patients with schizophrenia.

## Introduction

Cognitive deficits, including in aspects of attention, memory, learning, language, and executive function, are an essential feature of schizophrenia—being displayed in 98% of patients (Keefe et al., 2005; Tripathi et al., 2018). These cognitive deficits can impinge on patient prognosis and quality of life (Harvey et al., 2009). Previous evidence has shown that cognitive deficits in schizophrenia may be an innate trait, as they can occur in the initial episode as well as in patients with chronic schizophrenia (Gerretsen et al., 2017). Cognitive deficits can also occur in the prodromal stages and throughout the illness course, despite the alleviation of symptoms with pharmacological therapy (Hughes et al., 2003; Kahn and Keefe, 2013).

Although it is recognized that certain patients are predisposed to cognitive deficits, the underlying multi-faceted cause of cognitive deficits in schizophrenia remains largely obscure (Toulopoulou et al., 2007). However, accumulating evidence suggests a genetic contribution to cognitive deficits in schizophrenia patients (Harvey et al., 2016). For instance, cognitive deficits have even been observed in the unaffected relatives of schizophrenia patients (Snitz et al., 2006). Currently, a primary hypothesis is that neurodevelopmental and neurodegenerative dysfunction underlies the etiology of both cognitive deficits and schizophrenia (Li et al., 2020). The forkhead-box P2 (*FOXP2*) gene, located on chromosome 7q31, which contributes to brain development and neuronal processes, was first identified as being involved in language and speech (Lai et al., 2001). It is well established that disturbances of speech and language processes are central components of both schizophrenia and cognitive deficits (Li et al., 2009). Furthermore, *FOXP2* has been associated with schizophrenia in genome-wide association studies (Hagenaars et al., 2016). Our previous studies have reported that one of the *FOXP2* polymorphisms, rs10447760 within the 5’ regulatory region, is associated with clinical outcomes and cognitive deficits in chronic schizophrenia (Rao et al., 2017; Lang et al., 2019). Additionally, *FOXP2* has been associated with obesity in a genome-wide association study (Glessner et al., 2010; Clifton et al., 2018). Collectively, this evidence suggests that *FOXP2* contributes to both cognitive deficits and obesity in patients with schizophrenia.

In addition, it is now well established that critical, non-genetic aspects, such as metabolic syndrome and obesity, also contribute to cognitive deficits in schizophrenia patients (Rashid et al., 2013; Bora et al., 2017). Prior studies have also suggested a relationship between body mass index (BMI) and cognitive deficits (Smith et al., 2011). The frequency of higher BMI and obesity are greater in schizophrenic patients overall (Li et al., 2017), and the association of BMI with cognitive deficits in schizophrenia has also been established (Bora et al., 2017). However, other studies have reported inconsistent findings (Friedman et al., 2010; Takayanagi et al., 2012).

The above-mentioned inconsistent results may be due to the possible confounding interactions of sex, BMI, and genetic background. Increasing evidence has indicated that there are sex differences in clinical characteristics and outcomes, especially cognitive deficits, among schizophrenia patients across (Leger and Neill, 2016). Sex differences in brain structure and function, as well as sexually dimorphic steroid hormones, may contribute to the sex-specific effects on cognitive deficits in schizophrenic patients (Mendrek and Mancini-Marie, 2016). Previous studies have demonstrated different cognitive deficits between male and female patients with schizophrenia during their first and/or chronic episodes that were not observed in healthy controls (Leger and Neill, 2016; Mendrek and Mancini-Marie, 2016). However, the results are still equivocal. For example, several studies did not find an association between sex and cognitive deficits in patients with schizophrenia (Mendrek and Mancini-Marie, 2016).

Although our previous study has shown that *FOXP2* polymorphisms (rs10447760) is associated with cognitive deficits in chronic schizophrenia (Lang et al., 2019), it remains unclear whether there are sex differences in the degree of contribution of genetic factors to cognitive deficits in schizophrenia; whether there are sex differences in the influences of BMI on cognitive deficits in schizophrenia; and/or whether there are sex differences in the effects of genetic contributions and BMI on cognitive deficits in schizophrenia. These interesting questions prompt a need for further investigation.

Some previous evidence has demonstrated that sex differences in cognitive performance are associated with certain genetic backgrounds (Zhang et al., 2010; Jancke, 2018). Additionally, recent studies have shown that cognitive function and BMI may share a common genetic pathway (Davies et al., 2015; Marioni et al., 2016). Prior research has also suggested the existence of a shared genetic mutation that is associated with both BMI and cognitive performance (Marioni et al., 2016). Frazier-Wood et al. (2014) found that shared genetic factors contributed to cognitive function and BMI in a study of 1,312 twins. Additionally, Laitala et al. (2011) performed a study with a large sample of 2,606 twins which also showed a significant shared genetic influence on the correlation between cognitive decline and midlife BMI. The above evidence suggests that interactions between sex, genetic contribution, and BMI are involved in the pathophysiology of cognitive deficits.

Therefore, to the best of our knowledge, this study is the first to investigate: (1) whether the *FOXP2* polymorphism rs10447760 affects cognition differently as a function of sex; (2) whether BMI was associated with cognitive deficits in schizophrenia patients; (3) and whether any relationship between BMI and cognitive deficits in schizophrenia was further altered by sex.

## Materials and Methods

### Subjects

The protocol was approved by the ethics committee of the Beijing Huilongguan Hospital (No. BJ-7072035; Date: July 10th, 2016). Each subject or guardian signed a written informed consent prior to being enrolled in the study. The inclusion criteria have been described in previous studies (Lang et al., 2019), but, briefly, include: (1) patients must be between 18 and 75 years old; (2) patients must be of Han Chinese descent; (3) patients met diagnostic criteria for schizophrenia, as defined by the Diagnostic and Statistical Manual of Mental Disorders, Fourth Edition (DSM-IV); (4) patients experienced disease courses of at least 5 years; (5) patients had been treated mainly with monotherapy of an antipsychotic drug for at least 12 months.

Healthy controls without a family history of psychotic disorders were recruited through local advertisements. Individuals were excluded if they had any other major Axis I disorders.

Any patients who were pregnant or experiencing severe physical diseases, such as cardiovascular disease, cerebrovascular disease, cancer, infection, or unstable diabetes, were excluded. Any patient who had alcohol or drug abuse/dependence, as determined by laboratory urine tests, was excluded.

A total of 867 patients and 402 healthy controls were recruited. The demographic information of patients has been described in our previous studies. Briefly, there were differences in sex, BMI, and education between patients and healthy controls (Lang et al., 2019).

The antipsychotics that patients received have been described in our previous study but included clozapine, risperidone, quetiapine, chlorpromazine, sulpiride, aripiprazole, perphenazine, olanzapine, haloperidol, and other antipsychotics. The antipsychotic doses were calculated by being equivalent to chlorpromazine (Lang et al., 2019).

### Clinical interview assessments

All participants were interviewed independently by two psychiatrists using the Structured Clinical Interview for DSM-IV (SCID-I/P). We also used the Positive and Negative Symptoms Scale (PANSS) to assess clinical symptoms. Cognitive performance was evaluated using the Chinese version of the Assessment of Neuropsychological Status (RBANS, Form A).

### DNA isolation and SNP genotyping

Peripheral venous blood was sampled, and DNA was extracted using a salting-out method followed by preservation at −80^°^C (Rao et al., 2017).

The polymorphism rs10447760 of *FOXP2* was genotyped using the methods described before (Rao et al., 2017). 5% of the samples were randomly selected for repeated genotyping as a quality control measure.

### Statistical analyses

The normality of variable distribution was assessed using the Kolmogorov-Smirnov test. Chi-square tests and student’s *t*-tests were used, for categorical variables and continuous variables, respectively. Hardy–Weinberg equilibrium was detected using a goodness of fit of χ^2^ test.

To investigate the effects of differing antipsychotics on cognitive scores in patients, we conducted a multiple analysis of covariance (MANCOVA). We chose this approach to address the overall *p*-value because we thought it would reduce type I error, while also adjusting for age, BMI, smoking, education, onset age, illness duration, hospitalization times, antipsychotic duration, and daily dose. Then, an analysis of covariance (ANCOVA) was performed to examine the effects of different antipsychotics on the cognitive score, while adjusting for the same parameters as in the primary MANCOVA.

To identify the effects of sex on cognitive scores in patients and healthy controls, a 2 × 2 MANCOVA (sex × diagnosis) was conducted. All of the RBANS subscores were dependent variables, and diagnosis and sex were fixed factors. We also adjusted for age, BMI, smoking, and education level. Then, an ANCOVA was performed with individual RBANS scores as the dependent variable. In this model, sex and diagnosis were the fixed factors, and we adjusted for age, BMI, smoking, and education in the control group, and for onset age, hospitalization time, antipsychotic types (atypical or typical antipsychotics), duration of treatment with antipsychotics and antipsychotics dose in the patient group. Stepwise multivariate regression analyses were conducted with each RBANS score as a dependent variable, where sex was an independent variable, and the same confounding covariates were controlled for patients.

To identify the effects of the *FOXP2* genotype on the relationship between sex and cognitive scores in patients and healthy controls, we used 2 × 2 MANCOVAs (genotype × sex) separately in patient and control groups. All RBANS scores were set as dependent variables, with the genotype and sex as the independent variables, and adjustment for confounding covariates. We then used an ANCOVA with each RBANS score as a dependent variable, genotype, and sex as independent variables, and with separate adjustments for confounding covariates in the patient and control groups.

To explore the association between BMI and cognitive scores in the patients and controls separately, as well as in the different sex and genotypic groupings, we used partial correlations which adjusted for demographic and clinical covariates. We also carried out multivariate regression analyses, with each cognitive domain as a dependent variable, BMI as an independent variable, and controlling for confounding variables.

Bonferroni corrections were performed for multiple tests. The power of the sample in the present study was calculated using Quanto Software under dominant, log additive, and recessive models, and setting the prevalence of schizophrenia as 1% in the population. The statistical analyses were applied using SPSS version 25.0, and statistical significance was identified as a 2-tailed p-value that was less than 0.05.

## Results

### The effect of different antipsychotics on cognitive performance in patients

The MANCOVA demonstrated that there were no significant effects of differing antipsychotics on cognitive function (Wilks’ Lambda, *F* = 0.94, *p* = 0.61). Furthermore, the ANCOVA demonstrated that differing antipsychotics did not have different effects on immediate memory (*F* = 1.46, *p* = 0.16), visual space/structure (*F* = 0.85, *p* = 0.56), language (*F* = 0.51, *p* = 0.89), attention (*F* = 1.03, *p* = 0.42), delayed memory (*F* = 1.18, *p* = 0.31), or total RBANS scores (*F* = 1.13, *p* = 0.34).

### Sex difference in cognitive scores of patients and controls

The 2 × 2 MANCOVA analysis (sex × diagnosis), which adjusted for age, BMI, and educational years, showed a main effect of sex on cognitive performance (Wilks’ lambda *F* = 8.01; *p* < 0.0001). Then, as shown in Table 1, after adjustments for age, BMI, and educational years, ANCOVA showed a main effect of sex on language performance (*F* = 25.13; corrected *p* < 0.0001). There was a significant effect of diagnosis × sex on language scores (*F* = 16.71, *p* < 0.001) which also survived Bonferroni correction. Further ANCOVA analysis of the patient group showed male patients with schizophrenia had better language performance than female patients (*F* = 17.83; *p* < 0.0001), after adjusting for confounding variables. This result also persisted after Bonferroni correction (*p* < 0.0001). Further multivariate regression analysis demonstrated that sex was correlated with language performance in patients (*ß* = −7.69, *t* = 4.22, *p* < 0.0001); that is, female patients performed worse than males did. In the control group, there were no significant differences in any of the RBANS subscale scores, or the total RBANS scores, between males and females (*p* > 0.05).

**Table 1:** Comparisons among the RBANS total and five subscale scores by diagnostic and sex groupings (Mean ± SD).

Patients			Controls		Diagnosis		Sex		Diagnosis × Sex	
RBANS scores	Male (*N* = 684)	Female (*N* = 183)	Male (*N* = 158)	Female (*N* = 244)	F	p	F	p	F	p
Immediate memory	56.88 ± 15.58	60.02 ± 18.38	75.56 ± 15.79	75.77 ± 18.27	256.95	<0.001	3.69	0.06	0.17	0.68
Attention	64.19 ± 17.87	64.21 ± 20.45	89.28 ± 18.34	86.24 ± 21.52	420.86	<0.001	1.78	0.18	0.36	0.55
Language	76.11 ± 17.31^a^	69.25 ± 19.96^a^	95.07 ± 11.41	93.12 ± 14.04	444.23	<0.001	25.13	<0.001^b^	16.71	<0.001^a^
Visuospatial/construction	76.56 ± 18.23	76.86 ± 17.95	81.26 ± 15.61	78.55 ± 15.52	13.19	<0.001	1.93	0.17	0.04	0.85
Delayed memory	63.48 ± 19.09	66.72 ± 20.91	86.72 ± 14.24	85.94 ± 15.91	323.84	<0.001	0.24	0.62	0.04	0.85
Total score	60.88 ± 13.79	61.58 ± 16.66	80.64 ± 14.90	79.31 ± 16.12	436.776	<0.001	0.48	0.49	0.92	0.34

*^a^There was a significant effect of diagnosis X Gender on language score after a Bonferroni correction (*F* = 16.71, *p* < 0.001). Male patients had higher language performance than female patients. ^b^Furthermore, there was a significant effect of gender on language score after a Bonferroni correction (*F* = 25.13, *p* < 0.001). The language score was higher in male than in female patients with schizophrenia (*F* = 17.83; *p* < 0.0001), while no difference was found in language score between males and females in controls (*p* > 0.05)*.

### Sex differences in the effects of *FOXP2* rs10447760 on cognitive scores in patients and controls

The 2 × 2 MANCOVA demonstrated no effects of genotype (Wilks’ lambda *F* = 1.41; *p* = 0.21), sex (Wilks’ lambda *F* = 1.71; *p* = 0.12), or genotype × sex (Wilks’ lambda *F* = 0.56; *p* = 0.77) on cognitive performance. ANCOVA analysis showed that there were genotype effects on immediate memory scores (*F* = 4.14, *p* = 0.04) and language scores (*F* = 4.89, *p* = 0.03; Table 2) in the patient group. However, the results did not persist after Bonferroni correction (*p* = 0.24, *p* = 0.18). No sex differences in the genotypic effects on BMI was found.

**Table 2:** Comparisons among the RBANS total and five subscale scores by gender and genotypic groupings in patient group (Mean ± SD).

Male			Female		Sex		Genotype		Sex × Genotype	
RBANS scores	CC (*N* = 658)	CT (*N* = 26)	CC (*N* = 178)	CT (*N* = 5)	F	p	F	p	F	p
Immediate memory	57.05 ± 15.63	52.54 ± 14.00	60.45 ± 18.42	44.80 ± 8.04	0.003	0.96	4.14	0.04^a^	0.17	0.69
Attention	64.32 ± 18.00	60.96 ± 13.83	64.29 ± 20.58	61.40 ± 16.42	0.07	0.80	0.09	0.76	0.03	0.86
Language	76.19 ± 17.31	74.19 ± 17.53	69.50 ± 19.99	60.20 ± 18.21	4.89	0.03^a^	0.86	0.36	0.19	0.67
Visuospatial/construction	76.58 ± 18.27	75.92 ± 17.35	77.02 ± 17.93	71.20 ± 19.73	0.06	0.81	0.003	0.96	0.18	0.67
Delayed memory	63.50 ± 19.09	62.88 ± 19.33	66.95 ± 20.95	58.60 ± 19.41	0.02	0.88	0.09	0.77	<0.0001	0.99
Total score	60.97 ± 13.90	58.65 ± 10.76	61.79 ± 16.77	54.20 ± 10.92	0.23	0.63	0.86	0.36	0.02	0.90

*^a^The *p*-value did not survive after a Bonferroni correction*.

In the control group, there were no effects of sex, genotype, or sex × genotype on any of the RBANS scores (all *p* > 0.05).

### Sex difference in BMI of patients and healthy controls

There were no significant effects of sex or diagnosis × sex effects on BMI (*F* = 0.96, *p* = 0.33; *F* = 0.16, *p* = 0.69). Furthermore, there were no significant sex differences in BMI in any of the subjects, whether they were patients or healthy controls (all *p*’s > 0.05), which suggested that sex had no effects on BMI in patients or controls.

### Sex difference in the association between BMI and cognitive scores in patients and controls

To further investigate whether BMI was associated with cognitive scores in different sexes, we further divided the patients into a male patient group and a female patient group. Additional multivariate regression analysis demonstrated a positive association of BMI with language performance in the male patient group (*ß* = 0.60, *t* = 3.30, *p* = 0.001), while other cognitive subscale scores and total scores showed no significant differences after adjusting for confounding variables and performing Bonferroni corrections (all *p*’s > 0.05).

In the healthy control group, BMI was negatively correlated with attention performance in the male control group (*r* = −0.26, *p* = 0.001), including after controlling confounding covariables (partial *r* = -0.21, partial *p* = 0.009). However, this did not persist after Bonferroni correction. Other cognitive subscale scores and total scores showed no significant associations with BMI after adjusting for confounding variables (all *p*’s > 0.05).

### Sex differences in the association of BMI with cognitive performance: adjusted by *FOXP2* rs10447760

To further explore the association between BMI and cognitive performance in different *FOXP2* rs10447760 genotypes, we divided the patients into two genotypic groups: patients carrying CC and patients carrying CT. Multivariate regression analysis demonstrated a positive association between BMI and language scores in the patient group carrying the CC genotype (*ß* = 0.53, *t* = 3.16, *p* = 0.002). Interestingly, this positive association was found only in male patients (*ß* = 0.63, *t* = 3.44, *p* = 0.001), not in female patients (*p* > 0.05).

In the healthy control group, no association was found between BMI and any of the five RBANS subscale scores or total scores in either *FOXP2* genotypic subgroup, after adjusting for confounding variables and performing Bonferroni corrections (all *p*’s > 0.05).

## Discussion

The main findings of this study were: (1) male schizophrenia patients had better language performance than female patients; (2) the *FOXP2* rs10447760 genotype did not show an interaction with sex on cognitive performance in schizophrenia patients; (3) there was a positive correlation between BMI and language performance only in male schizophrenia patients; (4) after stratification by *FOXP2* rs10447760 genotype, there was a positive correlation between BMI and language performance only in schizophrenia patients carrying the CC genotype.

Considering differences in hormonal status, psychosocial stress, and brain structure and function between male and female patients with schizophrenia, previous evidence has documented sex-specific effects on cognitive deficits in schizophrenia (Han et al., 2012). However, these results are still controversial. Most prior studies have shown that male schizophrenia patients have relatively worse performances on various cognitive dimensions (Han et al., 2012; Leger and Neill, 2016), but some studies have shown opposite results (Gogos et al., 2010; Leger and Neill, 2016). Additionally, other studies have shown that sex has no effect on cognitive changes in schizophrenia (Karilampi et al., 2011; Kao et al., 2013). Our results demonstrated better language cognitive performance in males compared to female patients with chronic schizophrenia. Previously, Lewine et al. (1996) found greater deficits in verbal and spatial memory in female patients with schizophrenia. They also found the worse performance of the right hemisphere than that of the left hemisphere, consistent with previous evidence showing right-hemispheric brain activation contributes to language function (van Ettinger-Veenstra et al., 2010).

We have previously reported that *FOXP2* rs10447760 affects immediate memory in chronic schizophrenia (Lang et al., 2019). However, we did not find any effects of *FOXP2* rs10447760 on any cognitive scores across sexes, indicating that there were no interactive effects of *FOXP2* rs10447760 and sex on cognitive scores in schizophrenia. Few prior studies have explored the interplay of sex and gene polymorphism on cognitive deficits in schizophrenia. One previous study found significant sex interactions when assessing the relationship between BDNF AL66Met polymorphism, another neurotrophic factor, and cognitive performance (Kim et al., 2016). This evidence suggests that different genes, or even different polymorphisms in the same gene, can lead to different sex-specific interactions on cognitive functioning.

Higher BMI and obesity are common in schizophrenia patients. However, any effects of BMI on cognitive performance within schizophrenia patients are unknown (Bora et al., 2017). Previous findings have suggested that the influence of BMI on cognitive deficits in schizophrenia is complex (Friedman et al., 2010; Takayanagi et al., 2012; Rashid et al., 2013; Hidese et al., 2018). Other aspects, including sex and genetic background, may be involved in the association between BMI and cognitive deficits in schizophrenia patients (Rashid et al., 2013). A previous study by our group also showed that NRG3 polymorphism altered the effect of BMI on cognitive deficits of patients with schizophrenia (Zhou et al., 2020). Here, we showed that there was a positive association between BMI and language performance in schizophrenia patients, even after adjusting for demographic and clinical covariables. Interestingly, this positive association was found in male patients, but not in female patients. It seems likely that BMI affects cognitive deficits as a function of sex in schizophrenia. Previously, one study demonstrated that increased BMI correlated with worse executive function and attention in male patients with heart failure, but not in female patients, which also indicated an interacting sex effect on the relationship between BMI and cognitive function (Hawkins et al., 2014).

FOXP*2* plays a critical role in the neuronal processes that contribute to language and speech performance (Liegeois et al., 2003; Vernes et al., 2007), and its polymorphisms have been reported to be involved in the pathophysiology of cognitive function and language phenotypes in schizophrenia (Tolosa et al., 2010; Mozzi et al., 2017). However, we did not find any associations between the *FOXP2* rs10447760 genotype and language scores in our previous study (Lang et al., 2019), and a recent meta-analysis also showed no association between *FOXP2* variations and language deficits in schizophrenia (McCarthy et al., 2019). These inconsistent findings might be the result of foregoing consideration of other crucial demographic or clinical characteristics, including BMI and sex. In this study, although we did not observe an interactive effect between sex and *FOXP2* rs10447760 on cognitive deficits, we did find that *FOXP2* rs10447760 interplayed with BMI to affect cognitive changes in schizophrenia patients: There was a positive correlation of BMI with language function only in schizophrenia patients with the *FOXP2* rs10447760 CC genotype. This suggests that BMI might affect cognitive deficits in schizophrenia as a condition of specific *FOXP2* genotypes. *FOXP2* has also been associated with obesity in previous studies (Glessner et al., 2010; Xia and Grant, 2013; Clifton et al., 2018). Accumulating evidence indicates there is a shared genetic pathway or system for cognitive performance and BMI (Marioni et al., 2016). Marioni et al. (2016) demonstrated that individual genetic mutations correlating with BMI account for a critical component of the variance in cognitive performance, and *vice versa*. They also showed that genetic variants leading to higher cognitive performance correlated with lower BMI (Marioni et al., 2016). In addition, some studies have also suggested that genetic factors contribute highly to cognitive function and BMI phenotypes. For example, Frazier-Wood et al. (2014) reported between 20% and 30% shared genetic variance of cognitive function and BMI of 20%–30% (Laitala et al., 2011). Additionally, these shared genetic pathways or systems appear to display overlapping gene expression profiles in specific areas of the brain (Locke et al., 2015). Our present results provide further evidence of the shared genetic influences underlying BMI and cognitive function.

The present study has some limitations. First, we recruited patients with chronic schizophrenia and with different antipsychotic treatment regimens. However, several lines of evidence indicate that the style and severity of cognitive deficits are fundamentally stable. In addition, cognitive deficits were independent of illness duration and remained relatively stable without changes over time (Harvey et al., 1990; Bowie and Harvey, 2006). Furthermore, the different antipsychotics used all converted to chlorpromazine. Second, although we recruited relatively larger samples, unequal sample sizes between the patient and control groups, as we had here, may cause type II error. Furthermore, there were unequal sample sizes between males and females within patient or healthy controls, and unequal sample sizes between *FOXP2* CC and *FOXP2* CT genotypes within the patient group. Further studies in first episode drug naïve schizophrenia patients, and studies with equal sample sizes between patients and controls, should be conducted to validate our results. With regard to the unequal sample sizes between *FOXP2* C and T alleles within subjects, *FOXP2* rs10447760 has the rare variant allele T in the Asian population. Thus, a small number of subjects with CT genotype may dramatically change the significance of p-value. Last but not least, the differences in sex, BMI, and education between patients and healthy controls might influence the results, although these factors have been adjusted for.

In conclusion, the present study showed sex differences in language deficits in schizophrenia patients. Furthermore, sexual dimorphism and *FOXP2* rs10447760 may impact the influence of BMI on language-based cognitive deficits in schizophrenia patients.

## Data Availability Statement

The datasets presented in this study can be found in online repositories. The names of the repository/repositories and accession number(s) can be found below: https://ngdc.cncb.ac.cn/gsub/submit/bioproject/list,subSAM098688.

## Ethics Statement

The studies involving human participants were reviewed and approved and the protocol was approved by the ethics committee of the Beijing Huilongguan Hospital (No. BJ-7072035). The patients/participants provided their written informed consent to participate in this study.

## Authors Contributions

ZL, MXi, and XZ designed the study. ZL, MF, MXu, and YC wrote the article. MY and XL did the statistical analysis. All authors contributed to the article and approved the submitted version.

## Funding

This research was funded by National Natural Science Foundation of China (81401127, 62073058), Chengdu Municipal Health Commission (2021057), Open Project Program of State Key Laboratory of Virtual Reality Technology and Systems, Beihang University (No. VRLAB2022 B02), and Shanghai Key Laboratory of Psychotic Disorders Open Grant (21-K03). Funding had no role in study design, data analysis, or the decision to submit the article for publication.
